# The Role of Light–Dark Regulation of the Chloroplast ATP Synthase

**DOI:** 10.3389/fpls.2017.01248

**Published:** 2017-07-24

**Authors:** Kaori Kohzuma, John E. Froehlich, Geoffry A. Davis, Joshua A. Temple, Deepika Minhas, Amit Dhingra, Jeffrey A. Cruz, David M. Kramer

**Affiliations:** ^1^Department of Energy Plant Research Laboratory, Michigan State University, East Lansing MI, United States; ^2^Department of Biochemistry and Molecular Biology, Michigan State University, East Lansing MI, United States; ^3^Department of Cell and Molecular Biology, Michigan State University, East Lansing MI, United States; ^4^Department of Horticulture and Landscape Architecture, Washington State University, Washington DC, United States

**Keywords:** NPQ, ATP synthase, pmf, protein transport, twin arginine transporter, coupling factor, energy sensing, thioredoxin

## Abstract

The chloroplast ATP synthase catalyzes the light-driven synthesis of ATP and is activated in the light and inactivated in the dark by redox-modulation through the thioredoxin system. It has been proposed that this down-regulation is important for preventing wasteful hydrolysis of ATP in the dark. To test this proposal, we compared the effects of extended dark exposure in Arabidopsis lines expressing the wild-type and mutant forms of ATP synthase that are redox regulated or constitutively active. In contrast to the predictions of the model, we observed that plants with wild-type redox regulation lost photosynthetic capacity rapidly in darkness, whereas those expressing redox-insensitive form were far more stable. To explain these results, we propose that in wild-type plants, down-regulation of ATP synthase inhibits ATP hydrolysis, leading to dissipation of thylakoid proton motive force (pmf) and subsequent inhibition of protein transport across the thylakoid through the twin arginine transporter (Tat)-dependent and Sec-dependent import pathways, resulting in the selective loss of specific protein complexes. By contrast, in mutants with a redox-insensitive ATP synthase, pmf is maintained by ATP hydrolysis, thus allowing protein transport to maintain photosynthetic activities for extended periods in the dark. Hence, a basal level of Tat-dependent, as well as, Sec-dependent import activity, in the dark helps replenishes certain components of the photosynthetic complexes and thereby aids in maintaining overall complex activity. However, the influence of a dark pmf on thylakoid protein import, by itself, could not explain all the effects we observed in this study. For example, we also observed in wild type plants a large transient buildup of thylakoid pmf and nonphotochemical exciton quenching upon sudden illumination of dark adapted plants. Therefore, we conclude that down-regulation of the ATP synthase is probably not related to preventing loss of ATP *per se*. Instead, ATP synthase redox regulation may be impacting a number of cellular processes such as (1) the accumulation of chloroplast proteins and/or ions or (2) the responses of photosynthesis to rapid changes in light intensity. A model highlighting the complex interplay between ATP synthase regulation and pmf in maintaining various chloroplast functions in the dark is presented.

**Significance Statement:** We uncover an unexpected role for thioredoxin modulation of the chloroplast ATP synthase in regulating the dark-stability of the photosynthetic apparatus, most likely by controlling thylakoid membrane transport of proteins and ions.

## Introduction

Oxygenic photosynthesis stores energy in two chemical forms. Redox energy is stored by light-driven electron transfer reactions that result in the oxidation of water and the reduction of ferredoxin and NADPH. The electron transfer reactions are coupled to the translocation and deposition of protons into the thylakoid lumen, forming an electrochemical gradient of protons termed the proton motive force (pmf). The pmf, in turn, drives the reversible synthesis of ATP from ADP and inorganic phosphate (Pi), driven by the movement of protons from the thylakoid lumen to the stroma, catalyzed by the thylakoid CF_O_-CF_1_ ATP synthase (ATP synthase) (Junesch and Gräber, 1991; Seelert et al., 2003).

The ATP synthase is similar to its bacterial and mitochondrial orthologs (Okuno et al., 2011; Jonckheere et al., 2012) and is comprised of multiple subunits organized in two major sub-complexes, CF_O_ and CF_1_. The CF_O_ sub-complex consists of four subunits (I, II, III_14_, and IV, also called b, b’, c, and a); it couples trans-membrane proton translocation to the rotational motion of a ring of c subunits with respect to subunit a (Groth and Strotmann, 1999; Richter, 2004). The extrinsic CF_1_ subcomplex is made up of five subunits (α, β, γ, δ, and ε with stoichiometry of 3:3:1:1:1). The α and β subunits form a hexagonal α_3_β_3_ ring, housing three catalytic nucleotide-binding sites in the β subunits (Cross and Nalin, 1982; Abrahams et al., 1994). The γ subunit protrudes through the hexagon and is forced to rotate with respect to the ring, by action of CF_O_, imposing conformational distortions within the catalytic sites to drive ATP synthesis. The δ and ε subunits are proposed to stabilize the structure and regulate ATP hydrolysis activity (Richter et al., 2005).

The activity of the chloroplast ATP synthase is regulated by thioredoxin-mediated redox modulation of a pair of Cys residues on the γ**_1_** subunit (Schumann et al., 1985; Ort and Oxborough, 1992). Upon illumination, electron flow from photosystem I (PSI) reduces thioredoxin-f (TRX-f) via ferredoxin and ferredoxin:thioredoxin oxidoreductase (Schurmann and Buchanan, 2008) TRX-f, in turn, reduces a S-S bond on the γ subunit, resulting in a conformational change that lowers the threshold pmf required to activate the ATP synthase and leads to higher activity at low light (Fischer and Gräber, 1999). In the dark, the process is reversed when the γ-subunit is reoxidized, inactivating the complex.

It has been proposed that light/dark regulation of the ATP synthase serves to prevent the loss of ATP in darkness by proton transfer-linked ATP hydrolysis followed by proton leakage across the thylakoid (Ort and Oxborough, 1992). We term this hypothesis the “ATP conservation model.” Inactivating the ATP synthase by oxidation of its γ-subunit should prevent this energy leak. ATP synthase is rapidly activated at low light (∼4 μmol photons m^-2^ s^-1^) *in vivo*, owing to the relative high redox potential of its γ-subunit thiols (Kramer and Crofts, 1989; Kramer et al., 1990). In this way, thiol modulation could act as a digital “redox switch,” so that the ATP synthase is fully active even in low light, (preventing buildup of excessive pmf) and fully inactivated in the dark, preventing ATP loss. Consistent with this interpretation, Hisabori and coworkers (Sunamura et al., 2010) found a fractionally decreased cellular ATP content in the dark in a *Synechocystis* mutant with a modified ATP synthase γ-subunit that retained ATP hydrolysis activity in the dark. However, this mutation had no measureable effects on growth rates under light/dark cycle conditions, raising the question of the physiological impact of these effects. In contrast to higher plant chloroplasts, cyanobacteria are known to coexpress photosynthetic and respiratory pathways in the thylakoid membrane, and it is thus possible that ATP synthase activity is functionally maintained, even in darkness. We therefore aimed to test the ATP conservation model using mutants of Arabidopsis.

*Arabidopsis thaliana* possesses two ATP synthase γ subunit paralogs, designated γ**_1_** and γ**_2_**, coded by genes *ATPC1* and *ATPC2* (Inohara et al., 1991; Kohzuma et al., 2012). The existence of multiple γ-paralogs is not unique to Arabidopsis, and as of this writing, all dicots (but not monocots or green algae) thus far sequenced have at least two γ subunit paralogs that appear to have different regulatory properties (Kohzuma et al., 2012). In Arabidopsis, the γ**_1_** homolog is required for photosynthesis and confers on ATP synthase light-dark redox regulation. In contrast, γ**_2_** is mainly expressed in roots and appears to function in non-photosynthetic tissues. The Arabidopsis γ_2_-ATP synthase is redox insensitive and equally active in light and dark (Kohzuma et al., 2012). In this work, we took advantage of plants expressing either γ**_1_**- or γ**_2_**-ATP synthases to assess the possible function of redox down-regulation of the ATP synthase during extended dark exposure.

## Materials and Methods

### Plant Materials and Growth Conditions

Wild-type *Arabidopsis thaliana* (ecotype Wassileskija; Ws) and a series of mutants were grown on soil under continuous light period at 50 μmol photons m^-2^ s^-1^ at 22°C for 4 weeks, as described previously (Kohzuma et al., 2012). All experiments were performed on plants prior to bolting. Mutant lines included two independent lines, *gamera-1* and *gamera-2*, lacking *ATPC1 (dpa1*) but expressing ATPC2 under a 35S promoter (35S::*ATPC2).* A complemented line, *comp*, was generated by expressing 35S::*ATPC1* in the *dpa1* background (Dal Bosco et al., 2004). Similarly, the *minira-2* mutant, with low ATP synthase activity, was constructed by complementing *dpa1* with *ATPC1* gene containing a single point mutation in ATPC1 (P194M) under 35S control (35S::*ATPC1^P194M^)* according to Davis et al. (2016). This site-directed mutagenesis strategy was preferred over antisense suppression because of its relative stability over generational and developmental times.

For extended dark exposure, pots containing whole plants were enclosed in opaque boxes constructed from aluminum foil, including baffles to allow air flow, and incubated in the same growth chamber as described above. Extended dark treatments were performed for four days. The sixth and seventh leaves of each plant were taken at times indicated and used for RNA, Western blot and photosynthesis analysis.

For analysis of starch accumulation and breakdown, plants were grown under the same conditions as above, with the modification of changing the light treatment to a 16-hour/8-hour day/night cycle in order to measure the rates of starch degradation during the normal night period.

### *In Vivo* Spectroscopic Assays

Maximal PSII quantum efficiency (*F*_V_/*F*_M_), steady-state PSII quantum efficiency (Phi2), LEF, and ATP synthase activity (γ_H+_) were estimated as described (Kanazawa and Kramer, 2002; Cruz et al., 2005; Kohzuma et al., 2012). LEF values were calculated from the steady-state quantum efficiency (Φ_II_) parameter under 20-min steady-state exposure to 50 μmol photons m^-2^ s^-1^ photosynthetically actinic radiation.

### Protein Extraction and Western Blot Analyses

The relative contents of photosynthetic proteins in both wild-type and *gamera* were estimated using a Western blotting approach, as described previously (Kohzuma et al., 2012). Plant leaves were frozen and ground in liquid nitrogen, and proteins were extracted in 50 mM Tricine-KOH (pH7.5), 10 mM NaCl, 2 mM MgCl_2_, 10 mM EDTA, β-mercaptoethanol, and phenylmethylsulfonyl fluoride (PMSF). Chlorophyll determination was performed for each extract (Porra et al., 1989) and then all extracts were fractionated by centrifugation at 10,000 ×*g* for 10 min at 4°C into a soluble and membrane fraction. Recovered membrane fractions, containing essentially thylakoid membranes, were washed twice with extraction buffer and once with 80% acetone to remove pigments. Membrane fractions were loaded onto 12% SDS-PAGE gels based on equal chlorophyll content and separated. The resolved proteins were stained with either Coomassie or Ponceau red (when applicable) or transferred to polyvinyl difluoride membranes (Invitrogen, United States) for Western blot analysis. After transfer, proteins were immunodetected with specific polyclonal antibodies raised against the β subunit of ATP synthase (Agrisera, AS03-030); the D1 protein (Agrisera, AS01-016), OEC17 (Agrisera, AS06-142-16), OEC23 (Agrisera, AS06-167), OEC33 (Agrisera, AS05-092) of PSII; cytochrome (cyt) *f* (Agrisera, AS06-119), Rieske protein of the cytochrome *b*6*f* complex (Agrisera, AS08-330); and the F subunit (Agrisera, AS06-104) and D subunits of PSI (Agrisera, AS09-461) and dilutions used were according to the manufacturer specifactions (Agrisera^TM^) as described in Kohzuma et al. (2012). LHCII and Rubisco L subunit served as loading controls were detected by either Ponceau staining of Western blot membrane or Coomassie staining on gel, respectively.

### Imaging Analysis for Chlorophyll Fluorescence Measurements

Chlorophyll *a* fluorescence measurements were performed in a Dynamic Environmental Photosynthetic Imager (DEPI), as described in Attaran et al. (2014). Chambers were equipped with high-power 50W LEDs (Light emitting diodes) for white actinic illumination. Images corresponding to *F*_0_ and *F*_M_ were captured in the absence of actinic illumination and in the presence of a saturating actinic pulse (300 ms at 12,000 mmol photons m^-2^ s^-1^), respectively, by a CCD camera (AVT Manta 145 M) fitted with a near IR long pass filter (RT-830, Hoya Glass) and using a bank of Red LED (Luxeon Rebel, San Jose, CA, United States) to excite fluorescence. Images were processed using open source software (Schneider et al., 2012).

### Plasmids

The cDNA encoding Pea OEC17 (Oxygen Evolving Complex-23 kDa) and OEC33, (Cline et al., 1992) were cloned into pDEST14 (Invitrogen^®^) according to manufacture protocol.

### Isolation of Arabidopsis Thylakoids Used for Import Assays

Arabidopsis wild-type or *gamera* plants were grown on soil under 8 h light (100 μmol photons m^-2^ s^-1^)/16 h dark cycle at 22°C for 21–28 days. The chloroplasts were then isolated from these plants as essentially described by Aronsson and Jarvis (2002). Thylakoids were subsequently isolated as described by Bais and Schünemann (2011). Isolated thylakoids were washed two times in import buffer (50 mM HEPES-KOH, pH 8.0; 330 mM Sorbitol; IB) and then resuspended in import buffer to give a final concentration of 1 mg chlorophyll/ml. Thylakoids were constantly stored in the dark and on ice before use for targeting assays.

### Thylakoid Protein Targeting Assays Using *In Vitro* Translated Precursor Proteins

Pea OEC17 and OEC33 precursor proteins were radiolabeled using [3H]-Leucine and were translated using Promega’s TNT^®^ Coupled Reticulocyte Lysate System according to the manufacturer’s protocol. After translation, the radiolabeled PsOEC17 and PsOEC33 precursor proteins were diluted with an equal volume of ‘cold’ 50 mM L-Leucine in import buffer.

Thylakoid protein targeting assays were performed as described by Braun and Theg (2008) with the following specifications: A typical 150 μl reaction contained: 10 μl of either [^3^H] PsOEC17 or [^3^H]PsOEC33 precursor protein; 10 μl of either Ws or *gamera* thylakoids (60 μg chlorophyll/mL final concentration) and where indicated ATP (0.6 mM final concentration); dithiothreital (DTT) (1 mM final concentration); apyrase (10 units/ml final concentration); Tentoxin (12 μM) or Valinomycin/Nigericin (1 μM/0.5 μM respectively). Additionally, OEC33 imports were supplemented with 2X stromal extract as defined by Yuan and Cline (1994) Protein targeting assays were either performed in the light (100 μE) or in the dark for 20 min at 23°C and then terminated by centrifugation at 15,500 ×*g* on an Eppendorf microfuge for 5 min. The supernatant was removed and the thylakoid pellet was washed two times with import buffer and then finally pelleted. All thylakoid pellets were solubilized in 2X sample buffer (i.e., 120 mMTris-HCl(pH6.8); 4% SDS; 20% Glycerol; 0.02% Bromophenol Blue) and then analyzed by 12% Tricine SDS-PAGE. After electrophoresis, gels were subjected to fluorography and exposed to X-ray film (Eastman, Kodak, Rochester, NY, United States).

### Semi-quantitative RT-PCR

RNA was isolated from leaf tissue derived from wild-type and *gamera* plants incubated for extended dark conditions at 0, 2, and 4 days using an RNeasy Plant Mini kit according to protocol supplied by the manufacturer (Qiagen). Isolated RNA was quantitated using Aligent 2100 bioanalyzer. First-strand cDNA was synthesized from 1 μg of total RNA using SuperScript^TM^ III Reverse Transcriptase according to the manufacturer’s protocol (Invitrogen^TM^). PCR reactions were performed using various gene specific primers (Supplemental Table S1) while PCR amplification conditions were determined empirically for each template-primer pair (Supplemental Figures S3, S5). Specifically, the following general PCR conditions were used for all samples (Supplemental Figures S3, S5): Step 1: 95°C, 2 min; Steps 2–4: 95°C, 30 s; 55°C, 30 s; 68°C, 60 s; were steps 2–4 were repeated as empirically determined for each clone [see below]; Step 5: 68°C, 5 min; and finally Step 6: 4°C indefinitely. Cycle times for clones in Supplemental Figure S3 were as follows: SAG12, 27 cycles; SEN1, 22 cycles; RBCS2B and CAB2B, 19 cycles; and 18s rRNA, 14 cycles. Cycle times for clones in Supplemental Figure S4 were as follows: PsbA(D1 protein), 14 cycles; PsbQ (OEC16) and PsbP (OEC23), 27 cycles; PsbO (OEC33), 22 cycles; PetA (CytF), 20 cycles; PetC (Reiske protein), 24 cycles; PsaF, 22 cycles; PsaD2, 23 cycles; ATP-B, 19 cycles and 18s rRNA, 14 cycles.

### Leaf Starch Analysis

Visualization of leaf starch accumulation and breakdown was performed as follows: Both Ws and *gamera* were grown under a 16 h/8 h light cycle. Plants were stained with iodine to visualize starch accumulation 1-h before the end of the light cycle (the end of the day). Whereas, starch breakdown was similarly visualized 1-h before the end of the dark cycle (the end of the night). All leaves were bleached in 80% ethanol at 80°C and the starch content visualized by Lugol staining (0.34% I_2_, 0.68% KI).

## Results

### Photosynthesis in γ_2_-ATP Synthase (*gamera*) Mutants Is Highly Resistant to Extended Dark Exposure

**FIGURE 1:**
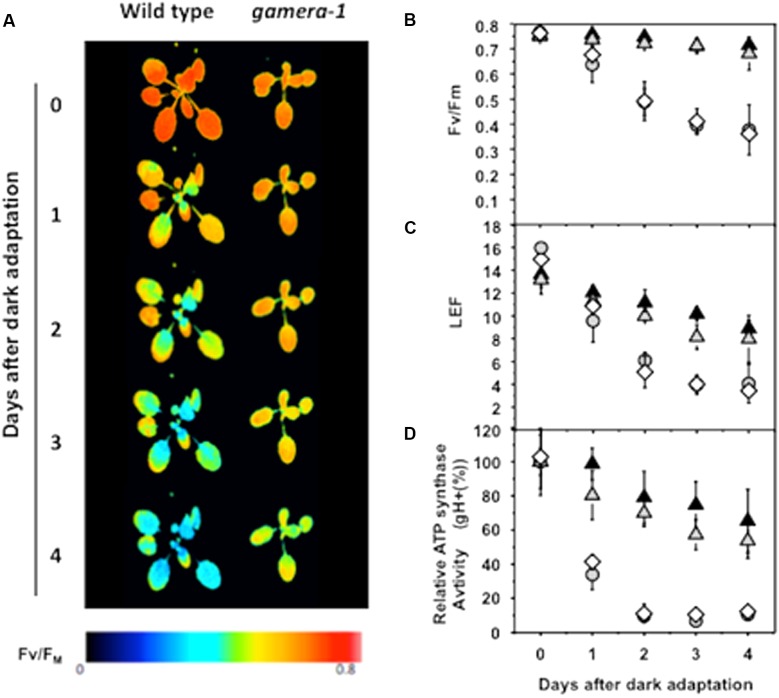
Effects of extended dark exposure on photosynthetic parameters in wild type (Ws) and mutants affecting ATP synthase regulation. Wild-type (Ws) (gray circles), 35S::ATPC1 in dpa1 (*comp*, white diamonds), *gamera*-1 (white gray triangles), and *gamera*-2 (black triangles) were subjected to extended dark exposure for four days. Illumination was minimized to prevent substantial activation of photosynthetic energy storage. A single saturation pulse was given for measurement of FV/FM, followed by 20 min 20 μmol photons m^-2^ s^-1^ illumination and a second saturation pulse for estimation of linear electron flow (LEF). **(A,B)** The maximum quantum yield of PSII (FV/FM) was estimated by chlorophyll a fluorescence imaging. The red and blue false coloring indicates high and low FV/FM values, as indicated in the key. **(C)** Light-driven linear electron flow (LEF) under steady-state illumination was calculated, based on chlorophyll a fluorescence parameters as described in Material and methods. **(D)** Fractional changes in the proton conductivity across the thylakoid membrane (gH+), estimated by the decay kinetics of the electrochromic shift (ECS), reflecting the activity of the chloroplast ATP synthase. The gH+ data was normalized to values at time zero, at which point Ws, 35S::ATPC1 in dpa1(comp), *gamera*-1 and *gamera*-2 showed relative gH+ values of 97.8 ± 7.6, 78.6 ± 2.1, 46.9 ± 7.3, and 41.0 ± 8.0 s-1, respectively. All measurements were averages of independent experiments on biological replicates (*n* = 4–5).

The ATP conservation model predicts that plants expressing redox-insensitive ATP synthases, which retain ATP hydrolysis activity in the dark, should be more sensitive to long dark exposure than wild type because of loss of ATP stores. In **Figure 1**, we compared several photosynthetic parameters during extended dark exposure for wild-type plants (ecotype Wassileskija-2, Ws), two independent lines in which γ_1_-ATP synthase expression was interrupted and replaced by 35S::ATPC2 (*gamera-1* and *gamera-2*), and a control line (*comp*) in which γ_1_-ATP synthase expression was interrupted, but replaced by 35S::*ATPC1*. More details on these lines can be found in Dal Bosco et al. (2004) and Kohzuma et al. (2012). Most work presented here is on *gamera-1*, with key experiments repeated on the independent line, *gamera-2.*

**Figure 1** shows that maximal quantum efficiency of PSII, estimated by the chlorophyll *a* fluorescence parameter *F*_V_/*F*_M_ in dark-adapted plants (**Figures 1A,B**), and linear electron flow (LEF, **Figure 1C**) were rapidly lost during extended dark in wild-type and *comp*. In contrast, *gamera* showed substantially *higher* stability, losing only a few percent of *F*_V_/*F*_M_ or f_II_ over four days. In addition, Ws and *comp* showed a strong loss of *in vivo* ATP synthase activity as estimated by the ECS decay kinetics parameter *g*_H_+ (Kanazawa and Kramer, 2002) during extended dark exposure, with over half of activity lost in 24 h (**Figure 1D**). In contrast, *gamera-1* and *gamera-2* mutants maintained substantially higher stability of ATP synthase activity, losing only 10–20% of activity over the first 24 h of dark exposure.

As a control, we also assessed the effects of extended dark exposure on *atpc2*, lacking ATPC2, but with wild-type ATPC1 (Supplemental Figure S1) (Kohzuma et al., 2012). The photosynthetic responses of *atpc2* were nearly identical to Col, consistent with previous work showing that in Ws, γ2-ATP synthase is not involved in photosynthesis (Kohzuma et al., 2012). In addition to its lack of redox regulation, the *gamera* mutants show an approximately 40% decrease in steady-state ATP synthase activity in the light (Kohzuma et al., 2012). To determine which of these effects was responsible for the extended dark stability of photosynthesis seen in *gamera*, we tested a site-directed mutant of the γ_1_-subunit, with a substitution of methionine at position 194, which is present in γ2, for proline (*atpc1^P194M^*, *minira-2*) (Davis et al., 2016). The *minira-2* line was generated to introduce amino acid changes found in the γ2 protein that are different from γ1 near the regulatory cysteines in order to understand the lack of redox regulation in *gamera* (Kohzuma et al., 2013; Davis et al., 2016). Like *gamera*, *minira-2* showed decreased ATP synthase activity during steady-state illumination (Supplemental Figure S2A), but retained wild-type-like light-dark redox regulation (Supplemental Figure S2B). No differences were observed between *minira-2* and Ws in *F*_V_/*F*_M_ measurements taken during extended dark exposure (Supplemental Figures S2C,D), indicating that decreased ATP synthase activity by itself did not affect the stability of PSII maximal quantum efficiency. We thus conclude that, in contrast to expectations from the ATP conservation model, eliminating redox down-regulation in *gamera-1* and *gamera-2*, increased rather than decreased the stability of the photosynthetic apparatus during extended dark exposure.

### Gradual Loss of Photosynthetic Activity in *gamera* during Extended Dark Is Not Related to Classical Senescence Regulation

We next tested the possibility that the gradual loss of photosynthetic activity in *gamera* was caused by activation of the classical plant senescence program, the hallmarks of which include chlorophyll degradation, loss of photosynthetic activity and increases in the expression of specific senescence genes (Weaver and Amasino, 2001; Bechtold et al., 2004; Keech et al., 2007; Wada et al., 2009; Yamatani et al., 2013). Expression levels in both light and dark of two classically inducible-senescence genes, *SAG12* and *SEN1* (Weaver et al., 1998) (Supplemental Figure S3A) and two senescence-sensitive nuclear encoded photosynthetic genes, *RBCS2* and *CAB2B*, were examined and the results showed that the trends between Ws and *gamera* plants were indistinguishable (Supplemental Figure S3A). Also, in contrast to the expected effects of senescence, chlorophyll content did not change during extended dark exposure in either Ws or *gamera* (Supplemental Figure S3B), indicating that *gamera*-induced changes in ATP synthase regulation did not affect classical senescence responses. We therefore conclude, that a senescence response could not account for the differences we observed in the photosynthetic behavior between Ws and *gamera* plants subjected to long-term dark treatment. Thus, other factors must be contributing to the down regulation of photosynthetic activity in both wild-type and *gamera* plants (See **Figure 1**).

### Differential Effects of *gamera* on Photosynthetic Proteins during Extended Dark Exposure

**FIGURE 2:**
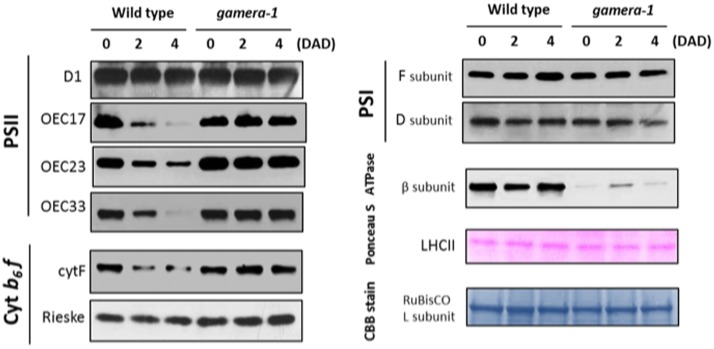
Changes in the protein levels of photosynthetic components under extended dark exposure in wild-type and *gamera-1*. Immunoblot detection of photosynthetic proteins from leaves of Ws and *gamera-1* plants incubated after dark adaptation for 0, 2, and 4 days was examined. Specifically, essentially thylakoid fractions were assayed to determine the content of the following proteins: β-subunit of ATP synthase; the D1 protein, OEC17, OEC23, and OEC33 of PSII; Cyt f and Rieske protein of the cytochrome b6f complex; and the F and D subunits of PSI, after extended dark treatment. Proteins were resolved via SDS-PAGE gel based on equal microgram chlorophyll per lane loading and processed as described in Section “Materials and Methods”. The Large subunit of RuBisco and LHCII stained with either CBB or Ponceau red, respectively, are presented here as loading controls. DAD indicates days after dark adaptation.

We further examined the effects of extended dark exposure on the levels of representative photosynthesis-related proteins using immunoblotting (**Figure 2**). In both Ws and *gamera*, the D1 protein of PSII and the F and D subunits of PSI appeared unchanged during extended dark period. By contrast, three subunits of the oxygen evolving complex (OEC), OEC17, OEC23, and OEC33, as well as, the cytochrome *f* protein of the cytochrome *b_6_f* complex declined substantially in Ws, but remained constant in *gamera*. In contrast, the Rieske protein of the cytochrome *b_6_f* complex remained constant for both Ws and *gamera* (**Figure 2**). Consequently, after subjecting plants to an extended dark treatment, the reduced levels of OEC proteins observed here would be expected to inactivate PSII complexes and thus could explain the reduction in *F*_V_/*F*_M_ in Ws seen in **Figure 1**. Finally, a disruption of the cytochrome *b_6_f* complex (i.e., by disrupting the accumulation of cyt *f*) would also result in diminished cytochrome *b_6_f* complex activity leading to decreased steady-state LEF (Schottler et al., 2007) which we observed in **Figure 1**.

Intriguingly, the observed effects on chloroplast protein levels we observed in **Figure 2** could not be explained merely by changes in gene expression. As shown in Supplemental Figure S4, the expression of several photosynthetic genes (*ATPB*, *PSBP*, *PETA*, *PSAF*, and *PSAD2*) showed similar decreases for Ws and *gamera* plants. In some cases, including *PSBQ, PSBO* and *PETC*, expression levels decreased more rapidly in *gamera* than in Ws. Finally, *PSBA* expression levels remained constant for both Ws and *gamera* plants. While our data shows no correlation between mRNA levels and protein levels (compare trends in **Figure 2** with Supplemental Figure S4), this type of result is not that uncommon (Vogel and Marcotte, 2012). With the advent of next-generation sequencing coupled with proteomics analysis, numerous studies have now revealed that mRNA levels quite often do not correspond to protein levels (Auld and Silver, 2006; Plotkin, 2010; Vogel and Marcotte, 2012). These studies point to a complex regulatory system for fine tuning protein levels through combinations of post-transcriptional, translational and degradation regulation. We conclude from the lack of correlation between the levels of mRNA (Supplemental Figure S4) and proteins (**Figure 2**) presented here that a ‘post-translational’ event is most likely controlling the disappearance of proteins during extended dark exposure.

### Evidence that ATP Synthase Regulation Affects Accumulation of Thylakoid Proteins by Controlling pmf Required for Protein Import in the Dark

Protein insertion into or transport across the thylakoid membrane can take place by at least four independent pathways, designated as Sec-dependent, SRP-dependent, ΔpH/Tat-dependent, and “spontaneous” (Schunemann, 2007; Cline and Dabney-Smith, 2008). In order to drive protein transport into the thylakoid lumen, a pmf is required for the ΔpH/Tat-dependent import pathway (Cline et al., 1992; Schubert et al., 2002; Braun et al., 2007; Braun and Theg, 2008), whereas, the Sec-dependent import pathway requires ATP and is stimulated by a ΔpH (Yuan and Cline, 1994; Mant et al., 1995).

In **Figure 2**, we show that after an extended dark period, components of the OEC complex (i.e. OEC33, OEC23 and OEC17) were significantly impacted. These components are transported into the thylakoid lumen using either the Tat- or Sec-dependent import pathways and thus require some level of a thylakoid pmf for their import, suggesting a mechanistic connection between ATP synthase activity, which should control thylakoid pmf in the dark, and the loss of thylakoid proteins. We hypothesized that the down-regulation of ATP synthase in the wild type should inhibit the generation of thylakoid pmf by ATP hydrolysis and thus inactivate Tat-dependent import, as well as, partially affect the Sec-dependent import pathway. As a consequence, the maintenance of the OEC complex and other Tat- and Sec-dependent complexes should be compromised during extended dark exposure, as we observed here (**Figure 2**). For example, the loss of OEC23 and 17 should have the added effect of decreasing the ‘stability’ of the OEC complex (Hashimoto et al., 1997; Ifuku et al., 2005; Yi et al., 2007, 2009; Ido et al., 2009; Kakiuchi et al., 2012). In a similar fashion, the down-regulation of ATP synthase activity during extended dark exposure could also have a wide-ranging impact on the stability of other photosynthetic complexes as well.

**FIGURE 3:**
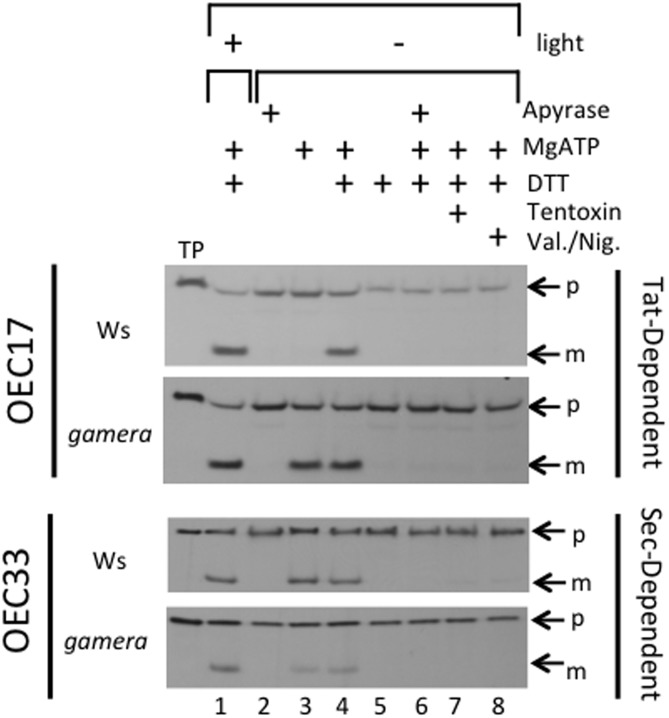
Effects of ATP synthase activity in wild-type and gamera on import of thylakoid Tat- and Sec-dependent proteins. The ability of ATP hydrolysis-driven proton translocation to drive the luminal import of either OEC17 (Tat-dependent) or OEC33 (Sec-dependent) into either wild-type or *gamera* thylakoids was assessed using protein import assays. Either [3H]OEC17 or [3H]OEC33 were incubated with isolated thylakoids from either wild-type or *gamera* plants in the presence (+) (lane 1) or absence (–) (lanes 2–8) of light at 23°C and, where indicated, with or without apyrase, ATP, DTT, Tentoxin, or Valinomycin/Nigericin for 20 minutes. TP, Translated protein: p, precursor protein; m, mature protein. OEC33 imports were supplemented with 2X stromal extract as defined by (Yuan and Cline, 1994). The figure shown is representative of one thylakoid import assay of three independent import assays performed.

To test this possibility, we assayed for effects of *gamera* on Tat-dependent protein import activities under reducing and oxidizing conditions in isolated thylakoids. For this import assay, we used the Tat-dependent substrate, OEC17 (Cline et al., 1992; Schubert et al., 2002; Braun et al., 2007; Braun and Theg, 2008), which is transported to the thylakoid lumen (**Figure 3**). Additionally, we also monitored the import of a Sec-dependent substrate, OEC33 which requires ATP and whose import is further stimulated by a ΔpH (Yuan and Cline, 1994; Mant et al., 1995). As shown in **Figure 3**, import of OEC17 was similar in Ws and *gamera* in the light, when LEF should maintain sufficient pmf to power the Tat machinery in both Ws and *gamera*. Under scrupulous dark adaptation when the γ-subunit should be oxidized, however, Ws thylakoids required both ATP and DTT to import OEC17 by Tat, reflecting the need for an activated ATP synthase complex to generate pmf by ATP hydrolysis. Our previous work demonstrated that the addition of DTT under these conditions led to reduction of the γ**_1_**-subunit thiols (Kohzuma et al., 2012). In contrast, *gamera* thylakoids required only ATP for active OEC17 transport under both oxidizing and reducing conditions, likely reflecting its redox-insensitive ATP synthase. We conclude from these results that γ_2_-ATP synthase in *gamera* is likely to remain active, even in the absence of thiol reductants, and thus may generate a sufficient pmf in the dark via ATP hydrolysis to support a basal level of thylakoid protein transport through Tat. In contrast, the wild type γ**_1_**-ATP synthase required activation of ATP synthase by DTT to support the Tat import pathway. By comparison, the Sec-dependent protein, OEC33, was imported into thylakoids regardless of the redox-state of ATP synthase (**Figure 3**). In addition, inhibition of ATP synthase function by Tentoxin treatment or dissipation of pmf by Valinomycin/Nigericin treatment prevented the import of OEC17, as well as, OEC33 into the thylakoid lumen (**Figure 3**). We thus propose, that the down-regulation of ATP synthase activity observed in wild type plants during extended dark periods, results in the dissipation of pmf that in time gradually reduces both Tat- and Sec-dependent import activity. As a result, the delicate balance between the turnover and replacement of various photosynthetic components during extended dark treatment becomes uneven culminating in the gradual inactivation of assorted photosynthetic complexes.

### Differential Degradation of Thylakoid Proteins in Ws and *gamera* Is Not Related to Starch Levels

Fixed carbon is stored as starch in the chloroplast during the day, and is broken down at night to supply energy for metabolic processes. Starch breakdown is highly regulated to maintain energy supplies over the length of the dark period (Sulpice et al., 2009). If starch is broken down more slowly in *gamera*, the chloroplast would be expected to retain sufficient energy to prevent degradation. We thus compared storage and depletion of starch in Ws and *gamera*. As shown in Supplemental Figure S5, both Ws and *gamera* showed starch accumulation in samples taken at the end of the light cycle. The lower starch storage in *gamera* was expected, based on the slower photosynthetic rates imposed by lower ATP synthase activities. Ws and *gamera* showed similar depletion of starch in the morning samples at the end of the night cycle, indicating that increased starch storage or decreased rates of breakdown could not account for the relative stability of the photosynthetic apparatus in *gamera*.

### Evidence that ATP Synthase Regulation Controls Transient NPQ during Rapid Dark/Light Transitions

**FIGURE 4:**
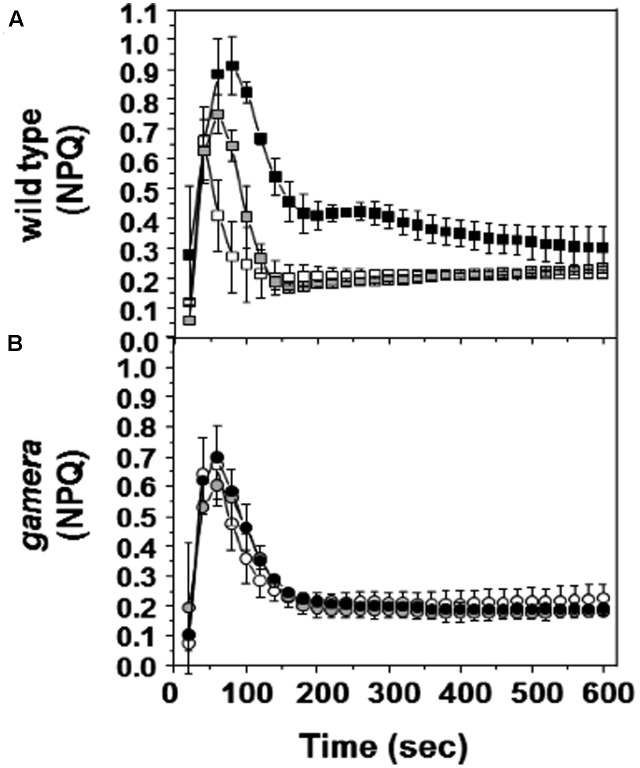
Effects of ATP synthase properties on transient NPQ. Induction kinetics of NPQ were measured upon abrupt light transition (from dark to 20 μmol photons m^-2^ s^-1^) after dark adaptation for 1 min (white square/circle), one hour (gray square/circle) and 10 h (black square/circle) in wild-type **(A)** and *gamera* **(B)**. Measurements are derived from several independent experiments (*n* = 4).

**Figure 4** shows the responses of NPQ during photosynthetic induction in Ws (**Figure 4A**) and *gamera* (**Figure 4B**), after varying times of dark adaptation. Both lines showed transient increases in NPQ, but the effect was strongly elevated and persisted longer in Ws as dark adaptation time was increased from a few minutes to an hour, i.e., the range when γ_1_ should be progressively more oxidized (Kramer and Crofts, 1989; Kramer et al., 1990). This effect likely reflects the activation of ATP synthase, which controls the buildup of pmf during the transition from darkness to light (Cardol et al., 2010). In Ws, dark-adaption leads to inactivation of ATP synthase and upon abrupt illumination, protons accumulate in the lumen, triggering a transient NPQ response that decreases as thiol modulation activates proton efflux through the ATP synthase. Consistent with this interpretation, the transient NPQ was smaller in *gamera*, probably because ATP synthase was already active before illumination.

## Discussion

### Possible Consequences for Photosynthetic Homeostasis

It has (very reasonably) been proposed that the chloroplast ATP synthase is inactivated in the dark to prevent loss of ATP through reversal of the normally light-driven process. However, it has not previously been possible to test this idea and (as discussed below) there may be other explanations for ATP synthase regulation.

We fully expected the mutant *gamera* that possesses an ATP synthase active in both the light and dark, to be more sensitive to extended dark periods because they would lose excessive amounts of ATP. However, *gamera* showed strikingly higher *stability* of photosynthetic activity during extended dark exposure (**Figures 1**–**3**). Immediately, these observations suggest that we should reassess the prevailing view that ATP synthase down-regulation is not required to prevent the loss of energy by ATP hydrolysis, leading to two very intriguing questions: How does *gamera* increase stability of photosynthesis? And what is the role of dark-induced degradation in the wild type?

While the differences in photosynthetic phenotypes (**Figure 1**) between wild-type and *gamera* grew progressively larger during extended dark exposure (**Figure 1**), significant differences were apparent even after one day, especially in the maximal PSII quantum efficiency (**Figures 1A,B**) and the activity of the chloroplast ATP synthase (**Figure 1D**). Extrapolating these effects to zero dark exposure reveals no signs of a lag period, suggesting that this dark-related loss of photosynthetic capacity occurs progressively (though perhaps subtly) during normal diurnal cycles. Thus, the loss of activity in the dark may reflect the operation of homeostatic mechanisms that normally fine tune photosynthesis under natural conditions, but become imbalanced due to exposure to extended dark periods.

There are many possible mechanisms by which *gamera* maintains photosynthesis in the dark, reflecting a range of possible functions. At this point, it is not possible to make clear conclusions, but based on our results and the existing literature, we may make some useful hypotheses.

We propose that one reason why *gamera* can maintain photosynthesis in the dark is due in part to the presence of an active pmf-dependent Tat import pathway, as well as, an active ‘pmf-stimulated’ Sec import pathway which in Ws appears to diminish over time. Consequently, in this scenario, interrupting thylakoid protein import in the dark could lead to loss of certain proteins in the lumen. This loss would, at first, affect the activities of complexes containing both Tat- and Sec-dependent proteins, but later could upset the regulation of many activities. Indeed, the potential accumulation of precursor proteins in the stroma could have diverse effects on the functioning of the chloroplast. For instance, these mistargeted proteins could begin performing secondary functions, acting as enzymes in different pathways in different compartments or performing non-catalytic “moonlighting functions”, for example, acting as signals to regulate gene expression. There is a growing list of such multifunctional proteins (Jeffery, 2009) in many organisms, and many cases are now documented in plants (Moore, 2004) including Hexokinase-1, which acts as sugar sensor to regulate a series of genes, mitochondrial hexokinase, which plays a role in plant cell apoptosis (Kim et al., 2006) chaperonins, which also regulate metabolic enzymes such as Rubisco (Vitlin Gruber et al., 2013), and Ferredoxin-dependent glutamate synthase, which plays a moonlighting role in sulfolipid biosynthesis by interacting with UDP-sulfoquinovose synthase (Shimojima et al., 2005). Hence, due to this unique possibility, we hypothesize that one potential consequence of a disrupted pmf-dependent Tat import pathway could be that proteins normally transferred to the lumen may be diverted to the stroma and thus begin performing unexpected moonlighting functions that result in diminished photosynthesis activity. As such, the ATP synthase may act as an unrecognized light and redox sensor.

Alternatively, other reasons besides the disruption of pmf-dependent Tat- and pmf-stimulated Sec- import activity could explain the loss of lumen proteins we observed in **Figure 2**. For instance, the gradual turnover of specific thylakoid translocation components themselves during extended dark treatment could explain the steady decrease of certain lumen proteins (**Figure 2**). Indeed, this possibility is supported by the observations of Voelker and Barkan (1995) who showed that mutations in the thylakoid translocation components, tha1 (SecA component) and hcf106 (Tat component), disrupted the localization of different sets of proteins destined for the thylakoid lumen. However, our study did not examine specifically the protein levels of various Tat- and Sec- pathway translocation components themselves. Nevertheless, one can envision that such reductions in these protein levels would have wide ranging effect on levels of various Tat- and Sec-dependent proteins within the thylakoids. Since, this is not what we observed, we propose that the regulation of the import activity of both the Tat- and Sec-dependent pathways best explains the drastic reductions of OEC17, OEC23 and OEC33 we saw in our dark extended treatment (**Figure 2**). However, this is not the complete story. We propose that the effect on Tat- and Sec- dependent import pathways (i.e., due to loss of translocation activity), by itself, could not explain all of the observed effects presented in this study (**Figure 2**), and it is likely that loss of pmf during extended dark treatment affects other processes needed to maintain the enzymatic activities of the photosynthetic apparatus.

One potential process that could be impacted by the loss of dark pmf in the wild type may involve the activation of enzymes involved in the ‘turnover and repair’ of various photosynthetic complexes. Significant work has already been done to elucidate the mechanism(s) by which different photosynthetic components undergo turnover and repair after high light exposure (Baena-González and Aro, 2002; Pokorska et al., 2009; Kato et al., 2012; Järvi et al., 2015). For example, it has been shown that the turnover of certain thylakoid membrane and lumen proteins is facilitated by the coordinated action of a family of thylakoid proteases, termed Deg and FtsH (Kley et al., 2011; Roberts et al., 2012; Schuhmann and Adamska, 2012). One member of the Deg family, Deg1, has been shown to be involved in the turnover of the D1 protein, OEC33 and plastocyanin (Roberts et al., 2012; Schuhmann and Adamska, 2012) while FtsH, in combination with various Deg proteases, is involved in D1 protein turnover (Chi et al., 2012). It has recently been demonstrated *in vitro* that Deg1 can become proteolytically active only when the thylakoid lumen becomes acidic (Kley et al., 2011). Intriguingly, in this study, we showed that after extended ‘dark’ exposure, the D1, PsaF, PsaD, and the Reiske proteins all remained stable in *gamera* (**Figure 2**), indicating that the pH-dependent activity of Deg1 could not directly account for the effects we observe (for one thing, we expect a more acidic lumen in *gamera*). Nevertheless, the behavior of Deg1 illustrates the principle that certain luminal enzymes are indeed regulated by pmf, and by extrapolation should be affected by the dissipation of pmf in the dark as ATP synthase is inactivated. Unexpectedly, the most dark-sensitive proteins we observed were components of the OEC and the cytochrome b_6_f complex, which were preferentially lost in Ws but were very stable in *gamera* (**Figure 2**). Currently, we do not know which protease(s) or by what mechanism(s) components of either the OEC complex or the cytochrome b_6_f complex are preferentially turned over during extended dark treatment. However, from our results, we hypothesize that possibly a unique pmf-dependent protease may be activated during extended darkness due to changes in pmf thus resulting in the gradual loss of lumen proteins.

Certainly, the slow disruption of the OEC complex as pmf changes in extended dark would have severe consequences for plants that must eventually encounter the dark/light transition. Hence, understanding and characterizing the factors involved in stabilizing the OEC during the dark period should be revealing. Indeed, to emphasize the OEC’s importance, several studies have already shown that the maintenance and stability of the OEC in particular is critical for the proper functioning of PSII. For instance, while investigating the biogenesis of the OEC complex, Hashimoto et al. (1997) demonstrated that a large pool of unassembled PSII extrinsic proteins (OEC 30, 23 and 17) that reside in the lumen of thylakoids are capable of eventually interacting and assembling with PSII. Consequently, they proposed that the pool of these unbound extrinsic proteins plays a critical role in the “maintenance of homeostasis with respect to turnover and assembly of PSII” (Hashimoto et al., 1997). Furthermore, various other studies have supported elements of this hypothesis and have shown that a reduction of a particular OEC protein can have a dramatic impact on the stability and efficiency of PSII function (Hashimoto et al., 1997; Ifuku et al., 2005; Yi et al., 2007, 2009; Ido et al., 2009; Kakiuchi et al., 2012). For example, Ifuku et al. (2005) using an RNAi approach to down-regulate PsbP (i.e., OEC23), demonstrated that PsbP, but not PsbQ (i.e., OEC17), is necessary for the normal function of PSII in higher plants *in vivo*. Further, Yi et al. (2007) have shown that reduced levels of PsbP in Psb-deficient plants resulted in the loss of PSII reaction center proteins. More recently, Allahverdiyeva et al. (2013) further showed that the reduction of the OEC components, PsbQ and PsbR, had a significant impact on such short-term regulatory mechanism as state transition and NPQ as a result of reduced PSII activity and unstable PSII super complexes. In a similar fashion, from our results, we propose that changes in pmf during extended dark periods appears to have a significant affect on “maintaining the protein homeostasis” of the thylakoid lumen and on the stability and turnover of some components of PSII. Hence mechanistically, we maintain that as components of the OEC gradual diminished during an extended dark period (**Figure 2**), the stability of the OEC becomes compromised resulting in reduced photosynthetic activity (**Figure 1**). Thus, the reduction of various OEC components that we observed in **Figure 2** during extended dark exposure reveals as yet another means by which pmf can impact photosynthetic function. Indeed, further investigations will be needed to characterize the unique pmf-related dark-dependent processes that are involved in stabilizing various photosynthetic components.

For instance, one such process that needs further investigation is the pmf-dependent mechanism whereby lumen homeostasis in the dark is achieved by means of fine-tuning the “composition” of pmf by ion counterbalancing. By managing pmf in this manner, plants can control a variety of thylakoid processes, including the import of ions critical for maintenance of photosynthetic complexes. One of many possible examples is Ca^2+^, which is a known cellular signal that responds to stresses and activates signal cascades in response to light-dark transitions (Sai and Johnson, 2002). In addition, Ca2+ is an essential cofactor for the functioning of the OEC and for maintaining photosynthesis activity. Consequently, Ca^2+^ must be accumulated in the lumen to the micromolar range (Lee and Brudvig, 2004), far from the nanomolar concentrations of free Ca^2+^ typically seen in most cells. There is strong evidence that a Ca^2+^ gradient is established by the activity of a thylakoid membrane transporter, which in turn is dependent on pmf (Ettinger et al., 1999). One may thus speculate that long-term down regulation of the ATP synthase in the dark may perturb pmf sufficiently to allow for the escape of Ca^2+^ from the lumen, triggering stromal Ca^2+^ spikes that leads to activation of various cellular processes. For instance, Chigri et al. (2005) have reported that calcium signaling seems to play a role in the regulation of import for a certain subset of nuclear-encoded chloroplast proteins. They propose that Ca^2+^ levels/signals helps regulate overall chloroplast protein content which is critical for the maintenance of photosynthesis as well as for the functioning of various metabolic pathways (Chigri et al., 2005). Consequently, drastic shifts/spikes in stromal Ca^2+^ levels due in part to lumen pmf changes can impact general protein targeting within chloroplasts. But, the interplay between pmf and Ca^2+^ levels can alter other cellular processes as well. For example, in addition to stromal Ca^2+^ spikes, we propose that the gradual loss of dark pmf over time may eventually decrease lumen Ca^2+^ levels to below the effective binding constant at the OEC thus lower its stability within the lumen and thereby causing a reduction in photosynthesis activity. Indeed, there is considerable evidence to support a Ca^2+^ -dependent role in maintaining OEC stability and activity. For example, it has been shown that PsbP and PsbQ interact to functionally optimize oxygen evolution at PSII. Coincidently, to perform this function both calcium and chloride are required (Miqyass et al., 2007; Popelkova and Yocum, 2007). In addition, it has been shown that the removal of PsbP and PsbQ by high salt treatment of PSII membranes results in a significant loss of oxygen evolution activity to about 25% of control. Interestingly, activity can be restored by returning reconstituted PsbP and PsbQ to extracted PSII in the presence of calcium and chloride (Ghanotakis et al., 1984, 1985; Miyao and Murata, 1984). Thus, PsbP and PsbQ appear to act in a coordinated manner along with calcium and chloride to ensure functional activity of PSII. Consequently, one can imagine that when a significant imbalance of pmf in the dark occurs ion fluxes are activated resulting in the disruption of PsbP/PsbQ interaction and the gradual loss of OEC stability which in turn reduces PSII activity.

Accordingly, from these and other observations, one can picture a more general mechanism by which various lumen as well as stromal homeostatic processes respond to changes in Ca^2+^ due to fluctuations in pmf which ultimately leads to changes in protein levels. Similar scenarios may be imagined for other ions or cofactors that depend on pmf for transport.

Finally, in addition to pmf impacting the stability of various photosynthetic complexes, we show that pmf modulation in the dark also plays a critical role in preparing the plant for the dark/light transition. For instance, from our results, we conclude that the activation of the ATP synthase is a key factor in the transient buildup of pmf that occurs during abrupt transitions from dark to light that result in electron transfer before the Calvin-Benson cycle is fully activated. We propose that this buildup of pmf slows light-driven electron transfer and activates NPQ (**Figure 4**), and thus ameliorates the accumulation of highly reactive, reduced electron carriers. On the other hand, high pmf may itself lead to photodamage (see Kramer et al., 1990). In any case, the effects on redox states and buildup of reactive intermediates may trigger regulatory phenomena (e.g., retrograde signaling) that influences photosynthetic homeostasis.

## Conclusion

**FIGURE 5:**
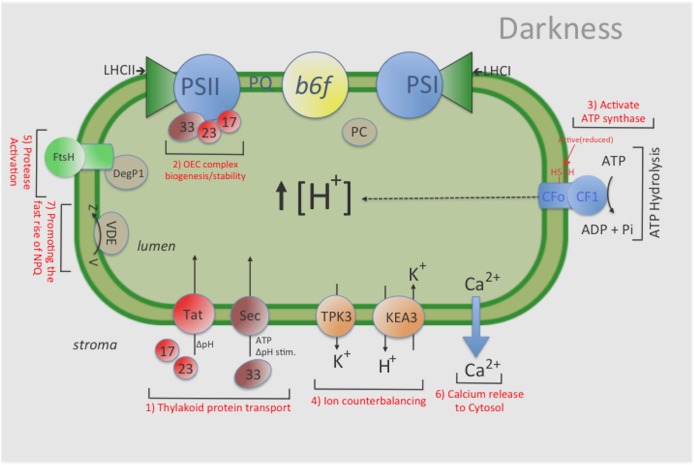
General overview of the influence that maintaining a ‘dark’ pmf has on various chloroplast functions. For example, a ‘dark’ pmf could: (1) provide the energy source for some level of protein import in the dark by the Tat-and Sec- dependent pathways, thus (2) maintaining an active photosynthesis apparatus (i.e., stable OEC complex); (3) help sustain a constitutive amount of activated (i.e., reduced) ATP-synthase complexes, thus enabling ATP synthesis upon illumination; (4) help sustain an overall ‘lumen’ homeostasis via ion counterbalancing which helps maintain the activities of various photosynthesis complexes; (5) modulate a low level of protease activity to remove damaged photosynthetic components; and (6) cause changes in Calcium levels in the lumen which could ultimately lead to changes in various proteins levels both in the thylakoid lumen and stroma and (7) promoting the fast rise of NPQ. Indeed, further investigations will be needed to better characterize and understand the role(s) that pmf plays in the overall photosynthetic process and in maintaining lumen homeostasis in the dark. KEA3: an electro-neutral K+ Efflux Antiporter 3; TPK3, the two-pore potassium (K+) channel 3.

We proposed that after wild type γ**_1_**-ATP synthase is turned off in the dark a sequence of events is activated to ensure that a ‘dark’ pmf is maintained and lumen homeostasis is both achieved and sustained. We further propose that maintenance of a dark pmf helps sustain some level of Tat-dependent, as well as, Sec-dependent import, which in turn replenishes certain components of the photosynthetic complexes and thereby aids in maintaining complex activity (**Figure 3**). However, the influence of pmf on overall protein import, by itself, cannot explain all the effects we observed in this study, and it is likely that loss of pmf affects other processes, including the transport of ions needed to maintain the activities of various photosynthetic complexes (See **Figure 5**; Checchetto et al., 2013; Hochmal et al., 2015). Indeed, further investigations will be needed to better characterize and understand the role(s) that pmf plays in the overall photosynthetic process and in maintaining lumen homeostasis in the dark.

## Author Contributions

KK, performed western blot, *in vivo* spectroscopic assays, various fluorescence experiments; JF, performed thylakoid import assays; GD, performed starch experiment; JT, performed Semi-quantitative RT-PCR; DM, AD, prepared various constructs; JC, performed imaging analysis for chlorophyll fluorescence measurements; DK, conceived project; KK, DK, and JF wrote portion of the manuscript.

## Conflict of Interest Statement

The authors declare that the research was conducted in the absence of any commercial or financial relationships that could be construed as a potential conflict of interest. The reviewer PP and handling Editor declared their shared affiliation, and the handling Editor states that the process met the standards of a fair and objective review.

## References

[B1] Abrahams J. P. Leslie A. G. Lutter R. Walker J. E. (1994). Structure at 2.8 A resolution of F1-ATPase from bovine heart mitochondria. *Nature* 370 621–628. 10.1038/370621a0 8065448

[B2] Allahverdiyeva Y. Suorsa M. Rossi F. Pavesi A. Kater M. M. Antonacci A. (2013). Arabidopsis plants lacking PsbQ and PsbR subunits of the oxygen-evolving complex show altered PSII super-complex organization and short-term adaptive mechanisms. *Plant J.* 75 671–684. 10.1111/tpj.12230 23647309

[B3] Aronsson H. Jarvis P. (2002). A simple method for isolating import-competent *Arabidopsis* chloroplasts. *FEBS Lett.* 529 215–220. 10.1016/S0014-5793(02)03342-2 12372603

[B4] Attaran E. Major I. T. Cruz J. A. Rosa B. A. Koo A. J. Chen J. (2014). Temporal dynamics of growth and photosynthesis suppression in response to jasmonate signaling. *Plant Physiol.* 165 1302–1314. 10.1104/pp.114.239004 24820026 PMC4081338

[B5] Auld K. L. Silver P. A. (2006). Transcriptional regulation by the proteasome as a mechanism for cellular protein homeostasis. *Cell Cycle* 5 1503–1505. 10.4161/cc.5.14.2979 16861887

[B6] Baena-González E. Aro E.-M. (2002). Biogenesis, assembly and turnover of photosystem II units. *Philos. Trans. R. Soc. Lond. B Biol. Sci.* 357 1451–1459. 10.1098/rstb.2002.1141 12437884 PMC1693054

[B7] Bais T. Schünemann D. (2011). Isolation of Arabidopsis thylakoid membranes and their use for in vitro protein insertion or transport assays. *Methods Mol. Biol.* 774 321–338. 10.1007/978-1-61779-234-2_19 21822847

[B8] Bechtold U. Murphy D. J. Mullineaux P. M. (2004). Arabidopsis peptide methionine sulfoxide reductase2 prevents cellular oxidative damage in long nights. *Plant Cell* 16 908–919. 10.1105/tpc.015818 15031406 PMC412865

[B9] Braun N. A. Davis A. W. Theg S. M. (2007). The chloroplast Tat pathway utilizes the transmembrane electric potential as an energy source. *Biophys. J.* 93 1993–1998. 10.1529/biophysj.106.098731 17513364 PMC1959559

[B10] Braun N. A. Theg S. M. (2008). The chloroplast Tat pathway transports substrates in the dark. *J. Biol. Chem.* 283 8822–8828. 10.1074/jbc.M708948200 18187420

[B11] Cardol P. De Paepe R. Franck F. Forti G. Finazzi G. (2010). The onset of NPQ and DmH+ upon illumination of tobacco plants studied through the influence of mitochondrial electron transport. *Biochim. Biophys. Acta* 1797 177–188. 10.1016/j.bbabio.2009.10.002 19836343

[B12] Checchetto V. Teardo E. Carraretto L. Formentin E. Bergantino E. Giacometti G. M. (2013). Regulation of photosynthesis by ion channels in cyanobacteria and higher plants. *Biophys. Chem.* 182 51–57. 23891570 10.1016/j.bpc.2013.06.006

[B13] Chi W. Sun X. Zhang L. (2012). The roles of chloroplast proteases in the biogenesis and maintenance of photosystem II. *Biochim. Biophys. Acta* 1817 239–246. 10.1016/j.bbabio.2011.05.014 21645493

[B14] Chigri F. Soll J. Vothknecht U. C. (2005). Calcium regulation of chloroplasts protein import. *Plant Cell* 42 821–831. 10.1111/j.1365-313x.2005.02414.x 15941396

[B15] Cline K. Dabney-Smith C. (2008). Plastid protein import and sorting: different paths to the same compartments. *Curr. Opin. Plant Biol.* 11 585–592. 10.1016/j.pbi.2008.10.008 18990609 PMC2628589

[B16] Cline K. Ettinger W. F. Theg S. M. (1992). Protein-specific energy requirements for protein transport across or into thylakoid membranes. Two lumenal proteins are transported in the absence of ATP. *J. Biol. Chem.* 267 2688–2696. 1733965

[B17] Cross R. L. Nalin C. M. (1982). Adenine nucleotide binding sites on beef heart F1-ATPase. Evidence for three exchangeable sites that are distinct from three noncatalytic sites. *J. Biol. Chem.* 257 2874–2881. 6460765

[B18] Cruz J. A. Avenson T. J. Kanazawa A. Takizawa K. Edwards G. E. Kramer D. M. (2005). Plasticity in light reactions of photosynthesis for energy production and photoprotection. *J. Exp. Bot.* 56 395–406. 10.1093/jxb/eri022 15533877

[B19] Dal Bosco C. Lezhneva L. Biehl A. Leister D. Strotmann H. Wanner G. (2004). Inactivation of the chloroplast ATP synthase gamma subunit results in high non-photochemical fluorescence quenching and altered nuclear gene expression in *Arabidopsis thaliana*. *J. Biol. Chem.* 279 1060–1069. 10.1074/jbc.M308435200 14576160

[B20] Davis G. A. Kanazawa A. Schöttler M. A. Kohzuma K. Froehlich J. E. Rutherford A. W. (2016). Limitations to photosynthesis by proton motive force-induced photosystem II photodamage. *Elife* 5:e16921 10.7554/eLife.16921 PMC5050024 27697149

[B21] Ettinger W. F. Clear A. M. Fanning K. J. Peck M. L. (1999). Identification of a Ca2^+^/H^+^ antiport in the plant chloroplast thylakoid membrane. *Plant Physiol.* 119 1379–1385. 10.1104/pp.119.4.1379 10198097 PMC32023

[B22] Fischer S. Gräber P. (1999). Comparison of ΔpH and Δψ driven ATP synthesis catalyzed by the H^+^-ATPases from *Escherichia coli* or chloroplasts reconstituted into liposomes. *FEBS Lett.* 457 327–332. 10.1016/S0014-5793(99)01060-1 10471802

[B23] Ghanotakis D. F. Babcock G. T. Yocum C. F. (1984). Calcium reconstitutes high rates of oxygen evolution in polypeptide depleted photosystem II preparations. *FEBS Lett.* 167 127–130. 10.1016/0014-5793(84)80846-7

[B24] Ghanotakis D. F. Babcock G. T. Yocum C. F. (1985). On the role of water soluble-polypeptides (17.23 kDa), calcium and chloride in photosynthetic oxygen evolution. *FEBS Lett.* 192 1–3. 10.1016/0014-5793(85)80030-23915890

[B25] Groth G. Strotmann H. (1999). New results about structure, function and regulation of the chloroplast ATP synthase (CFoCF1). *Physiol. Plant.* 106 142–148. 10.1034/j.1399-3054.1999.106120.x

[B26] Hashimoto A. Ettinger W. F. Yamamoto Y. Theg S. M. (1997). Assembly of newly imported oxygen-evolving complex subunits in isolated chloroplasts: sites of assembly and mechanism of binding. *Plant Cell* 9 441–452. 10.1105/tpc.9.3.441 12237359 PMC156929

[B27] Hochmal A. K. Schulze S. Trompelt K. Hippler M. (2015). Calcium-dependent regulation of photosynthesis. *Biochim. Biophys. Acta* 1847 993–1003. 10.1016/j.bbabio.2015.02.010 25687895

[B28] Ido K. Ifuku K. Yamamoto Y. Ishihara S. Murakami A. Takabe K. (2009). Knockdown of the PsbP protein does not prevent assembly of the dimeric PSII core complex but impairs accumulation of photosystem II supercomplexes in tobacco. *Biochim. Biophys. Acta* 1787 873–881. 10.1016/j.bbabio.2009.03.004 19285950

[B29] Ifuku K. Yamamoto Y. Ono T. A. Ishihara S. Sato F. (2005). PsbP protein, but not PsbQ protein, is essential for the regulation and stabilization of photosystem II in higher plants. *Plant Physiol.* 139 1175–1184. 10.1104/pp.105.068643 16244145 PMC1283756

[B30] Inohara N. Iwamoto A. Moriyama Y. Shimomura S. Maeda M. Futai M. (1991). Two genes, atpC1 and atpC2, for the gamma subunit of *Arabidopsis thaliana* chloroplast ATP synthase. *J. Biol. Chem.* 266 7333–7338. 1826905

[B31] Järvi S. Suorsa M. Aro E.-M. (2015). Photosystem II repair in plant chloroplasts — Regulation, assisting proteins and shared components with photosystem II biogenesis. *Biochim. Biophys. Acta* 1847 900–909. 10.1016/j.bbabio.2015.01.006 25615587

[B32] Jeffery C. J. (2009). Moonlighting proteins–an update. *Mol. Biosyst.* 5 345–350. 10.1039/b900658n 19396370

[B33] Jonckheere A. I. Smeitink J. A. Rodenburg R. J. (2012). Mitochondrial ATP synthase: architecture, function and pathology. *J. Inherit. Metab. Dis.* 35 211–225. 10.1007/s10545-011-9382-9 21874297 PMC3278611

[B34] Junesch U. Gräber P. (1991). The rate of ATP-synthesis as a function of ΔpH and Δψ catalyzed by the active, reduced H^+^-ATPase from chloroplasts. *FEBS Lett.* 294 275–278. 10.1016/0014-5793(91)81447-G 1661688

[B35] Kakiuchi S. Uno C. Ido K. Nishimura T. Noguchi T. Ifuku K. (2012). The PsbQ protein stabilizes the functional binding of the PsbP protein to photosystem II in higher plants. *Biochim. Biophys. Acta* 1817 1346–1351. 10.1016/j.bbabio.2012.01.009 22306528

[B36] Kanazawa A. Kramer D. M. (2002). In vivo modulation of nonphotochemical exciton quenching (NPQ) by regulation of the chloroplast ATP synthase. *Proc. Natl. Acad. Sci. U.S.A.* 99 12789–12794. 10.1073/pnas.182427499 12192092 PMC130538

[B37] Kato Y. Sun X. Zhang L. Sakamoto W. (2012). Cooperative D1 degradation in the photosystem II repair mediated by chloroplastic proteases in Arabidopsis. *Plant Physiol.* 159 1428–1439. 10.1104/pp.112.199042 22698923 PMC3425188

[B38] Keech O. Pesquet E. Ahad A. Askne A. Nordvall D. Vodnala S. M. (2007). The different fates of mitochondria and chloroplasts during dark-induced senescence in *Arabidopsis* leaves. *Plant Cell Environ.* 30 1523–1534. 10.1111/j.1365-3040.2007.01724.x 17986154

[B39] Kim M. Lim J. H. Ahn C. S. Park K. Kim G. T. Kim W. T. (2006). Mitochondria-associated hexokinases play a role in the control of programmed cell death in *Nicotiana benthamiana*. *Plant Cell* 18 2341–2355. 10.1105/tpc.106.041509 16920781 PMC1560927

[B40] Kley J. Schmidt B. Boyanov B. Stolt-Bergner P. C. Kirk R. Ehrmann M. (2011). Structural adaptation of the plant protease Deg1 to repair photosystem II during light exposure. *Nat. Struct. Mol. Biol.* 18 728–731. 10.1038/nsmb.2055 21532594

[B41] Kohzuma K. Dal Bosco C. Kanazawa A. Dhingra A. Nitschke W. Meurer J. (2012). Thioredoxin-insensitive plastid ATP synthase that performs moonlighting functions. *Proc. Natl. Acad. Sci. U.S.A.* 109 3293–3298. 10.1073/pnas.1115728109 22328157 PMC3295299

[B42] Kohzuma K. Dal Bosco C. Meurer J. Kramer D. M. (2013). Light- and metabolism-related regulation of the chloroplast ATP synthase has distinct mechanisms and functions. *J. Biol. Chem.* 288 13156–13163. 10.1074/jbc.M113.453225 23486473 PMC3642356

[B43] Kramer D. M. Crofts A. R. (1989). Activation of the chloroplast ATPase measured by the electrochromic change in leaves of intact plants. *Biochim. Biophys. Acta* 976 28–41. 10.1016/S0005-2728(89)80186-0

[B44] Kramer D. M. Wise R. R. Frederick J. R. Alm D. M. Hesketh J. D. Ort D. R. (1990). Regulation of coupling factor in field-grown sunflower: a Redox model relating coupling factor activity to the activities of other thioredoxin-dependent chloroplast enzymes. *Photosynth. Res.* 26 213–222. 10.1007/BF00033134 24420586

[B45] Lee C.-I. Brudvig G. W. (2004). Investigation of the functional role of Ca^2+^ in the oxygen-evolving complex of photosystem II: a pH-dependence study of the substitution of Ca^2+^ by Sr^2+^. *J. Chin. Chem. Soc.* 51 1221–1228. 10.1002/jccs.200400178

[B46] Mant A. Schmidt I. Herrmann R. G. Robinson C. Klösgen R. B. (1995). Sec-dependent thylakoid protein translocation. Delta pH requirement is dictated by passenger protein and ATP concentration. *J. Biol. Chem.* 270 23275–23281. 10.1074/jbc.270.40.23275 7559481

[B47] Miqyass M. van Gorkom H. J. Yocum C. F. (2007). The PS II calcium site revisited. *Photosynth. Res.* 92 275–287. 10.1007/s11120-006-9124-2 17235491

[B48] Miyao M. Murata N. (1984). Calcium ions can be substituted for the 24 kDa polypeptide in photosynthetic oxygen evolution. *FEBS Lett.* 168 118–120. 10.1016/0014-5793(84)80218-5

[B49] Moore B. (2004). Bifunctional and moonlighting enzymes: lighting the way to regulatory control. *Trends Plant Sci.* 9 221–228. 10.1016/j.tplants.2004.03.005 15130547

[B50] Okuno D. Iino R. Noji H. (2011). Rotation and structure of FoF1-ATP synthase. *J. Biochem.* 149 655–664. 10.1093/jb/mvr049 21524994

[B51] Ort D. R. Oxborough K. (1992). In situ regulation of chloroplast coupling factor activity. *Annu. Rev. Plant Physiol. Plant Mol. Biol.* 43 269–291. 10.1146/annurev.pp.43.060192.001413

[B52] Plotkin J. B. (2010). Transcriptional regulation is only half the story. *Mol. Syst. Biol.* 6 406 10.1038/msb.2010.63 PMC2950086 20739928

[B53] Pokorska B. Zienkiewicz M. Powikrowska M. Drozak A. Romanowska E. (2009). Differential turnover of the photosystem II reaction centre D1 protein in mesophyll and bundle sheath chloroplasts of maize. *Biochim. Biophys. Acta* 1787 1161–1169. 10.1016/j.bbabio.2009.05.002 19450540

[B54] Popelkova H. Yocum C. F. (2007). Current status of the role of Cl- ion in the oxygen-evolving complex. *Photosynth. Res.* 93 111–121. 10.1007/s11120-006-9121-5 17200880

[B55] Porra R. J. Thompson W. A. Kriedemann P. E. (1989). Determination of accurate extinction coefficients and simultaneous equations for assaying chlorophylls *a* and *b* extracted with four different solvents: verification of the concentration of chlorophyll standards by atomic absorption spectroscopy. *Biochem. Biophys. Acta* 975 384–394. 10.1016/s0005-2728(89)80347-0

[B56] Richter M. L. (2004). Gamma-epsilon interactions regulate the chloroplast ATP synthase. *Photosynth. Res.* 79 319–329. 10.1023/B:PRES.0000017157.08098.36 16328798

[B57] Richter M. L. Samra H. S. He F. Giessel A. J. Kuczera K. K. (2005). Coupling proton movement to ATP synthesis in the chloroplast ATP synthase. *J. Bioenerg. Biomembr.* 37 467–473. 10.1007/s10863-005-9493-9 16691485

[B58] Roberts I. N. Lam X. T. Miranda H. Kieselbach T. Funk C. (2012). Degradation of PsbO by the Deg protease HhoA Is thioredoxin dependent. *PLoS ONE* 7:e45713 10.1371/journal.pone.0045713 PMC3446894 23029195

[B59] Sai J. Johnson C. H. (2002). Dark-stimulated calcium ion fluxes in the chloroplast stroma and cytosol. *Plant Cell* 14 1279–1291. 10.1105/tpc.000653 12084827 PMC150780

[B60] Schneider C. A. Rasband W. S. Eliceiri K. W. (2012). NIH Image to ImageJ: 25 years of image analysis. *Nat. Methods* 9 671–675. 10.1038/nmeth.2089 22930834 PMC5554542

[B61] Schottler M. A. Flugel C. Thiele W. Bock R. (2007). Knock-out of the plastid-encoded PetL subunit results in reduced stability and accelerated leaf age-dependent loss of the cytochrome b6f complex. *J. Biol. Chem.* 282 976–985. 10.1074/jbc.M606436200 17114182

[B62] Schubert M. Petersson U. A. Haas B. J. Funk C. Schröder W. P. Kieselbach T. (2002). Proteome map of the chloroplast lumen of *Arabidopsis thaliana*. *J. Biol. Chem.* 277 8354–8365. 10.1074/jbc.M108575200 11719511

[B63] Schuhmann H. Adamska I. (2012). Deg proteases and their role in protein quality control and processing in different subcellular compartments of the plant cell. *Physiol. Plant.* 145 224–234. 10.1111/j.1399-3054.2011.01533.x 22008015

[B64] Schumann J. Richter M. L. McCarty R. E. (1985). Partial proteolysis as a probe of the conformation of the gamma subunit in activated soluble and membrane-bound chloroplast coupling factor 1. *J. Biol. Chem.* 260 11817–11823. 2864336

[B65] Schunemann D. (2007). Mechanisms of protein import into thylakoids of chloroplasts. *Biol. Chem.* 388 907–915. 10.1515/BC.2007.111 17696774

[B66] Schurmann P. Buchanan B. B. (2008). The ferredoxin/thioredoxin system of oxygenic photosynthesis. *Antioxid. Redox Signal.* 10 1235–1274. 10.1089/ars.2007.1931 18377232

[B67] Seelert H. Dencher N. A. Muller D. J. (2003). Fourteen protomers compose the oligomer III of the proton-rotor in spinach chloroplast ATP synthase. *J. Mol. Biol.* 333 337–344. 10.1016/j.jmb.2003.08.046 14529620

[B68] Shimojima M. Hoffmann-Benning S. Garavito R. M. Benning C. (2005). Ferredoxin-dependent glutamate synthase moonlights in plant sulfolipid biosynthesis by forming a complex with SQD1. *Arch. Biochem. Biophys.* 436 206–214. 10.1016/j.abb.2005.02.005 15752726

[B69] Sulpice R. Pyl E. T. Ishihara H. Trenkamp S. Steinfath M. Witucka-Wall H. (2009). Starch as a major integrator in the regulation of plant growth. *Proc. Natl. Acad. Sci. U.S.A.* 106 10348–10353. 10.1073/pnas.0903478106 19506259 PMC2693182

[B70] Sunamura E. Konno H. Imashimizu-Kobayashi M. Sugano Y. Hisabori T. (2010). Physiological impact of intrinsic ADP inhibition of cyanobacterial FoF1 conferred by the inherent sequence inserted into the gamma subunit. *Plant Cell Physiol.* 51 855–865. 10.1093/pcp/pcq061 20421199

[B71] Vitlin Gruber A. Nisemblat S. Azem A. Weiss C. (2013). The complexity of chloroplast chaperonins. *Trends Plant Sci.* 18 688–694. 10.1016/j.tplants.2013.08.001 24035661

[B72] Voelker R. Barkan A. (1995). Two nuclear mutations disrupt distinct pathways for targeting proteins to the chloroplast thylakoid. *EMBO J.* 14 3905–3914. 7664731 10.1002/j.1460-2075.1995.tb00062.x PMC394469

[B73] Vogel C. Marcotte E. M. (2012). Insights into the regulation of protein abundance from proteomic and transcriptomic analyses. *Nat. Rev. Genet.* 13 227–232. 10.1038/nrg3185 22411467 PMC3654667

[B74] Wada S. Ishida H. Izumi M. Yoshimoto K. Ohsumi Y. Mae T. (2009). Autophagy plays a role in chloroplast degradation during senescence in individually darkened leaves. *Plant Physiol.* 149 885–893. 10.1104/pp.108.130013 19074627 PMC2633819

[B75] Weaver L. M. Amasino R. M. (2001). Senescence is induced in individually darkened Arabidopsis leaves, but inhibited in whole darkened plants. *Plant Physiol.* 127 876–886. 10.1104/pp.010312 11706170 PMC129259

[B76] Weaver L. M. Gan S. Quirino B. Amasino R. M. (1998). A comparison of the expression patterns of several senescence-associated genes in response to stress and hormone treatment. *Plant Mol. Biol.* 37 455–469. 10.1023/A:1005934428906 9617813

[B77] Yamatani H. Sato Y. Masuda Y. Kato Y. Morita R. Fukunaga K. (2013). *NYC4*, the rice ortholog of Arabidopsis *THF1*, is involved in the degradation of chlorophyll - protein complexes during leaf senescence. *Plant J.* 74 652–662. 10.1111/tpj.12154 23432654

[B78] Yi X. Hargett S. R. Frankel L. K. Bricker T. M. (2009). The PsbP protein, but not the PsbQ protein, is required for normal thylakoid architecture in *Arabidopsis thaliana*. *FEBS Lett.* 583 2142–2147. 10.1016/j.febslet.2009.05.048 19500580

[B79] Yi X. Hargett S. R. Liu H. Frankel L. K. Bricker T. M. (2007). The PsbP protein is required for photosystem II complex assembly/stability and photoautotrophy in *Arabidopsis thaliana*. *J. Biol. Chem.* 282 24833–24841. 10.1074/jbc.M705011200 17604269

[B80] Yuan J. Cline K. (1994). Plastocyanin and the 33-kDa subunit of the oxygen-evolving complex are transported into thylakoids with similar requirements as predicted from pathway specificity. *J. Biol. Chem.* 269 18463–18467. 8034593

