# Supplementary material for: The Role of Light–Dark Regulation of the Chloroplast ATP Synthase
Source: Front Plant Sci. 2017 Jul 24;8:1248. doi: 10.3389/fpls.2017.01248 (PMC5522872; doi:10.3389/fpls.2017.01248)
Supplement: Supplementary file 1 [file Presentation_1.pdf]

Supplemental data

Supplemental Figure S1. Effects of extended dark exposure on photosynthetic parameters in Col and *atpc2*, lacking the ATP synthase γ_2 protein.

Supplemental Figure S2. The effects of reduced ATP synthase activity (g_{H+}) but not redox regulation on the stability of maximal PSII quantum efficiency during extended dark exposure.

Supplemental Figure S3. The effects of extended dark exposure on the expression of senescence-related genes and chlorophyll content.

Supplemental Figure S4. Expression of representative photosynthesis-related genes under extend dark conditions at 0, 2 and 4 days in wild-type (Ws) and *gamera*-1.

Supplemental Figure S5. Visualization of leaf starch accumulation and breakdown.

Supplemental Table S1. Gene Specific Primer sets used for RT-PCR.

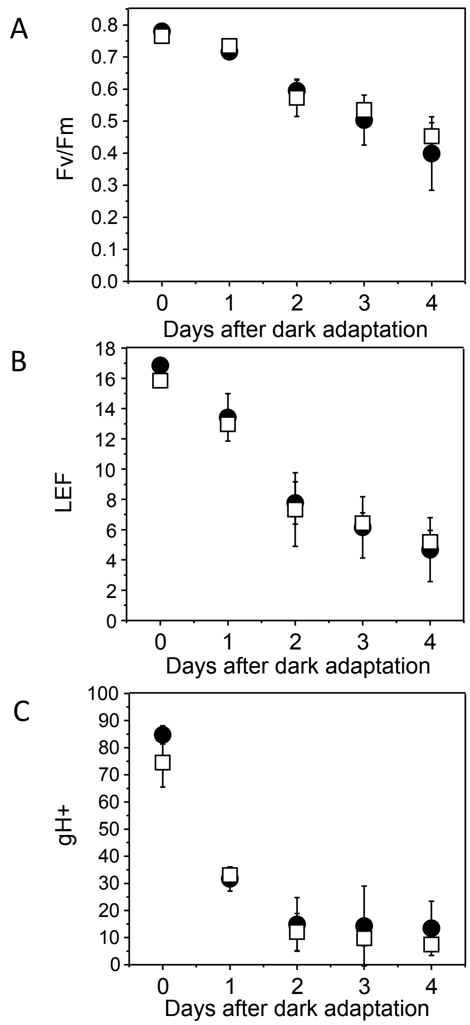

Supplemental Figure S1. Effects of extended dark exposure on photosynthetic parameters in Col and *atpc2*, lacking the ATP synthase γ_2 protein. Extended dark exposure and photosynthetic measurements were carried as in Main Text Fig. 1, out for 4 days in wild-type (Ws) (black circles), and *atpc2* (white squares). (**A**) Spectroscopy analysis were performed at different actinic light intensities – dependence of F_V/F_M, (**B**) Linear electron flow (LEF), (**C**) proton conductivity across the thylakoid membrane (g_{H+}) based on ECS decay kinetics. Data presented was the average and standard deviation of results from experiments on 4-5 biological replicates.

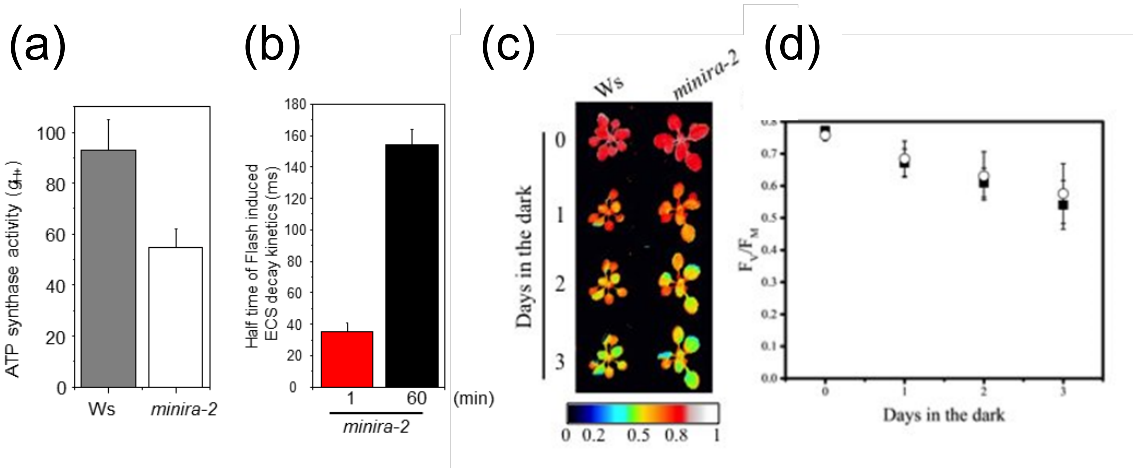

Supplemental Figure S2. The effects of reduced ATP synthase activity (g_{H+}) but not redox regulation on the stability of maximal PSII quantum efficiency during extended dark exposure. A construct containing a single point mutation between *ATPC1/ATPC2*, P194M (*minira-2*), was made and used to complement *dpa1*, which lacks *ATPC1*. The steady-state ATP synthase activity as estimated by the dark interval relaxation kinetics of the ECS (Ws-gray bar and *minira-2* white bar) showed a decrease of 41% activity from Ws and *minira-2* at 200 µmol photons m^{-2} s^{-1} PAR (**A**), similar to the loss of ATP synthase activity in *gamera* (Kohzuma et al. 2012). However, the decay of the flash-induced ECS signals increased upon exposure from 1 (red bar) to 60 (black bar) min of darkness (**B**), indicating wild type-like redox regulation of the ATP synthase (Kramer and Croft 1989). Measurements of F_V/F_M were taken once a day during three days of continuous darkness in a DEPI chamber using chlorophyll visual imaging (C) and quantified as described in Figure 1 of the main text (d, n=3). No differences were seen in the loss of F_V/F_M values over three days in darkness indicating that loss of ATP synthase activity by itself does not alter the decay of PSII during extended darkness (**D**).

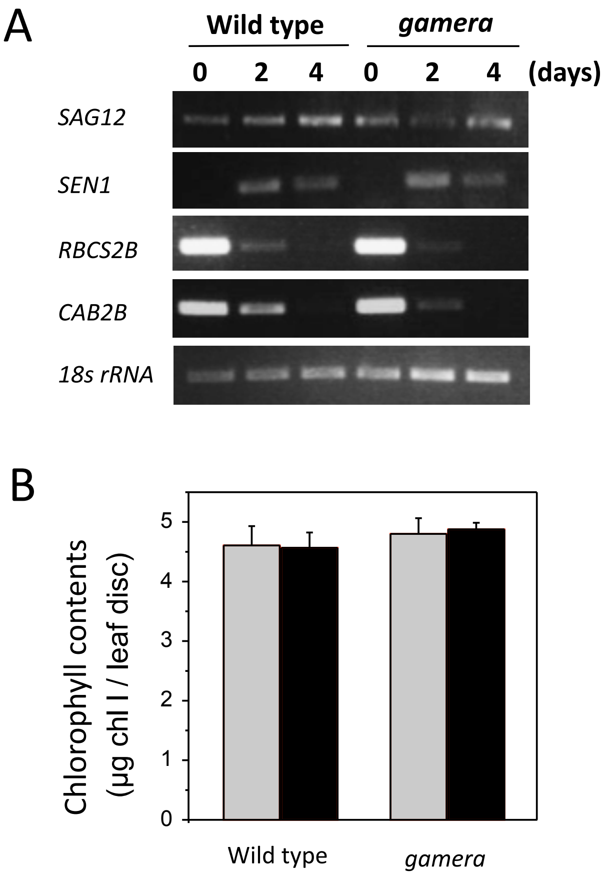

Supplemental Figure S3. The effects of extended dark exposure on the expression of senescence-related genes and chlorophyll content. Wild-type (Ws) and *gamera*-1 plants were treated as in Fig. 1 and assayed after 0, 2 and 4 days of darkness. (**A**) RT-PCR was performed to monitor expression levels of two classical senescence marker genes, *SAG12* and *SEN1*, as well as two photosynthetic genes, *RBCS2B* and *CAB2B*. 18s rRNA expression level was used as an internal loading control. For more details regarding RT-PCR conditions see Materials and Methods. (**B**) Chlorophyll content was extracted and determined from 6mm diameter leaf discs derived from wild-type and *gamera* plants at 0 day (gray bar) and after 4 days of dark adaption period (dark bar). Reported are averages and standard deviations of 4 biological replicates.

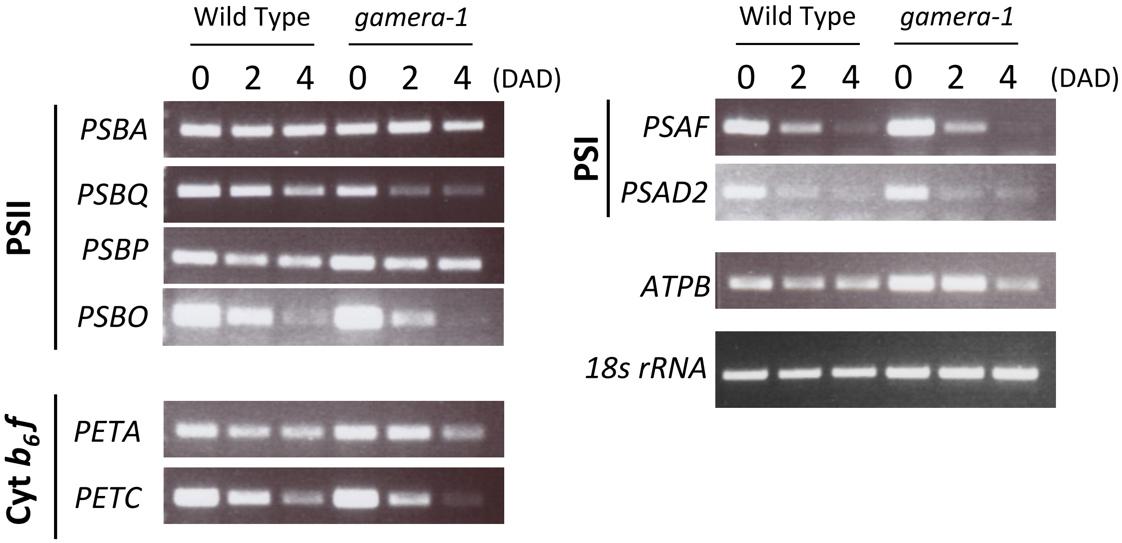

Supplemental Figure S4. Expression of representative photosynthesis-related genes under extend dark conditions at 0, 2 and 4 days in wild-type (Ws) and *gamera*-1. RT-PCR was performed using gene specific primers (Primers sets are listed in Supporting Table S1). PCR amplification conditions were determined empirically for each template-primer pair. 18s rRNA was used as an internal standard. For more details regarding RT-PCR conditions see Materials and Methods. Similar results were obtained among three independent experiments.

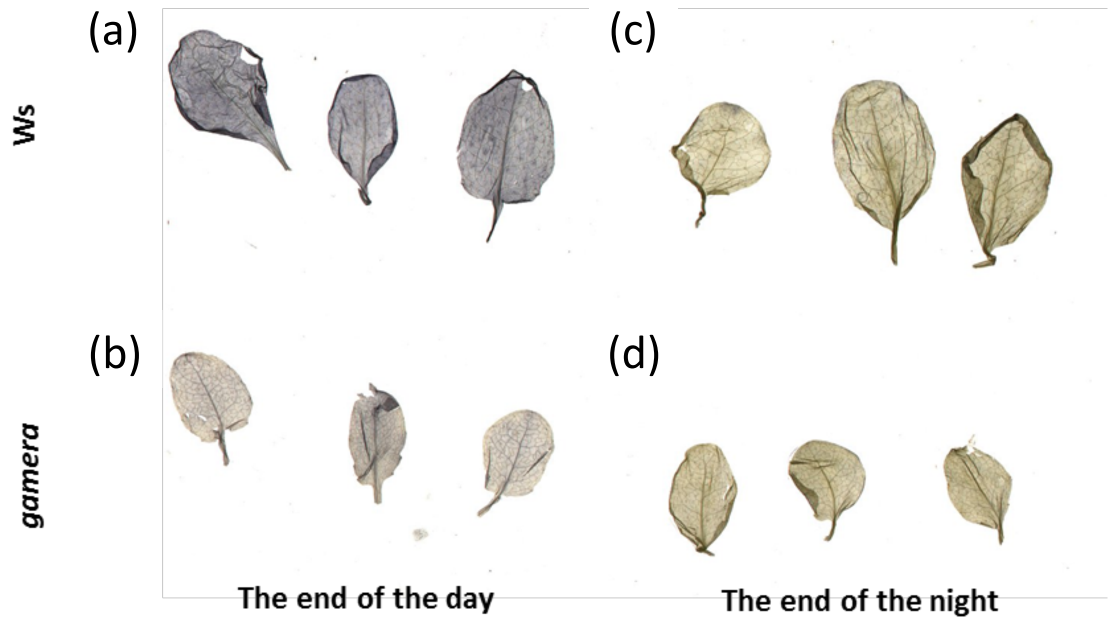

Supplemental Figure S5. Visualization of leaf starch accumulation and breakdown. Plants grown under a 16 hr/8 hr light cycle were stained with iodine to visualize starch accumulation 1-hour before the end of the light cycle (the end of the day) in Ws (**A**) and *gamera* (**B**). Starch breakdown was similarly visualized 1-hour before the end of the dark cycle (the end of the night) in Ws (**C**) and *gamera* (**D**). The lack of iodine staining in the end of the night samples is expected due to the normal breakdown of starch reserves by the end of the normal night period.

Supplemental Table S1. Gene Specific Primer sets used for RT-PCR.

RNA was isolated from leaf tissue derived from wild-type and *gamera* plants incubated for extended dark conditions at 0, 2 and 4 days using an RNeasy Plant Mini kit according to protocol supplied by the manufacturer (Qiagen). Isolated RNA was quantitated using Aligent 2100 bioanalyzer. First-strand cDNA was synthesized from 1μg of total RNA using SuperScript™III Reverse Transcriptase according to the manufacturer's protocol (Invitrogen™). PCR reactions were performed using various gene specific primers (Table S1) while PCR amplification conditions were determined empirically for each template-primer pair (Supplemental Figure. S3 and Supplemental Figure. S5).

Gene Name	At ID#	Primer Direction	Sequence	Fragment Length (bp)
SAG12	At5g45890	Forward	5'-GATGAAGGCAGTGGCACACCAA-3"	333
		Reverse	5'-TCCCACACAAACATACACAATTAAAAGC-3'	
SEN1	At4g35770	Forward	5'-ATCACGAATTGGAAACTGG-3'	133
		Reverse	5'-CTTTCCTCCATCGGAAG-3'	
RBCS2B	At5g38420	Forward	5'-ACCTTCTCCGCAACAAGTGG-3'	256
		Reverse	5'-GTGAAGCTTGGGGGCTTGTAGG-3'	
CAB2B	At1g29920	Forward	5'-TTGAAGGCTACAGAGTCGCAGGAAA-3'	290
		Reverse	5'-CACTCACGAAGCAAAGACTGAAGCA-3'	
18srRNA	-------------	Forward	5'-AACGGCTACCACATCCAAG-3'	442
		Reverse	5'GAAGCCAACACAATAGGAT-3'	
ATPB	AtCg00480	Forward	5'-GTAGCAAGAGCACGAAAAAT-3'	210
		Reverse	5'-TAAGTTCGTAGCCTTCGCAG-3'	
PSBA	AtCg00020	Forward	5'-CAATATGCTAGTTTCAACAA-3'	200
		Reverse	5'-GTTCATGCATAACTTCCATA-3'	
OEC17	At4g05180	Forward	5'-TAGATTTTACATACAACCAT-3'	250
		Reverse	5'-GTCCAAGTTGTCAATGGTTT-3'	
OEC23	At2g30790	Forward	5'-AGCAACTGCAAACATTTTGG-3'	150
		Reverse	5'-ATGTAAAGCTTTCCACCATT-3'	
OEC33	At5g66570	Forward	5'-ACTTCCTTCACCGTCAAGGC-3'	200
		Reverse	5'-TGGAAGTTGGACTGTGACTG-3'	
PETA	AtCg00540	Forward	5'-ATGGACGCGAAGTTATTGAT-3'	240
		Reverse	5'-AAGTACTATTTCCGCATCCC-3'	
PETC	At4g03280	Forward	5'-AGGGAGATCCGACTTACCTA-3'	200
		Reverse	5'-AGCCAACGCTAGCGACAATG-3'	
PSAF	At1g31330	Forward	5'-GTTTACCGCACTTGATAGTG-3'	200
		Reverse	5'-ACCACGGAAGATGATCCGAC-3'	
PSAD2	At1g03130	Forward	5'-AAGTACAAGATCACTTACCA-3'	180
		Reverse	5'-TTGTTTCCCAGTGAATTTAA-3'	
